# Modified Stability of microRNA-Loaded Nanoparticles

**DOI:** 10.3390/pharmaceutics14091829

**Published:** 2022-08-30

**Authors:** Katja Fresacher-Scheiber, Ivana Ruseska, Henrik Siboni, Martin Reiser, Fabio Falsone, Leonhard Grill, Andreas Zimmer

**Affiliations:** 1 Institute of Pharmaceutical Sciences, Department of Pharmaceutical Technology and Biopharmacy, University of Graz, Universitätsplatz 1, 8010 Graz, Austria; 2 Institute of Chemistry, Department of Physical Chemistry, University of Graz, Heinrichstraße 28, 8010 Graz, Austria

**Keywords:** protamine, microRNA, citric acid, nanoparticles, functionalization, binding affinity

## Abstract

microRNAs represent promising drugs to treat and prevent several diseases, such as diabetes mellitus. microRNA delivery brings many obstacles to overcome, and one strategy to bypass them is the manufacturing of self-assembled microRNA protein nanoparticles. In this work, a microRNA was combined with the cell-penetrating peptide protamine, forming so-called proticles. Previous studies demonstrated a lack of microRNA dissociation from proticles. Therefore, the goal of this study was to show the success of functionalizing binary proticles with citric acid in order to reduce the binding strength between the microRNA and protamine and further enable sufficient dissociation. Thus, we outline the importance of the present protons provided by the acid in influencing colloidal stability, achieving a constant particle size, and monodispersing the particle size distribution. The use of citric acid also provoked an increase in drug loading. Against all expectations, the AFM investigations demonstrated that our nanoparticles were loose complexes mainly consisting of water, and the addition of citric acid led to a change in shape. Moreover, a successful reduction in binding affinity and nanoparticulate stability are highlighted. Low cellular toxicity and a constant cellular uptake are demonstrated, and as uptake routes, active and passive pathways are discussed.

## 1. Introduction

RNA interference (RNAi) is one of the most remarkable findings in the past 25 years. In this endogenous process, small non-coding RNAs, such as microRNAs (miRNAs) or small interfering RNAs (siRNAs), post-transcriptionally regulate gene expression by binding to the complementary messenger RNAs (mRNA) [1,2]. Therefore, as RNAi has unique roles in regulating the functions and stability of mRNAs, it has become a promising and attractive treatment alternative for several metabolic and immunological disorders [3,4]. In particular, miRNA replacement or miRNA inhibition therapy are of great interest. Unlike exogenous siRNAs, endogenous miRNAs are not administered as therapeutic agents to this point. Thus, synthetic miRNAs mimicking the representation of miRNA duplexes, which contain the guide strand of the complementary miRNAs, are introduced [5,6]. Unfortunately, nucleic acid-based therapeutics face several major obstacles, including enzymatic degradation, insufficient membrane permeability, and low intracellular release [7]. Therefore, the development of adequate drug delivery systems (DDSs) to carry these modern biological active drugs to their targets and provide successful drug release is gaining more and more momentum [8,9]. Nanoparticles (NPs) formed via self-assembling represent attractive candidates for dealing with these challenges [10,11]. Furthermore, they are easily produced and do provide a myriad of engineering possibilities [12].

Cell-penetrating peptides (CPPs) are widely used to incorporate oligonucleotides (ODNs) in NPs [13]. These peptides—also known as protein translocation domains—are molecules able to bypass the limitations of conventional therapeutics and deliver therapeutic macromolecules [7]. One huge advantage of CPPs is their noninvasive manner of entering cells [14]. Additionally, the cytotoxicity of CPPs is referred to as very low, and they do not show any immunological responses [14,15,16]. This work will focus on a CPP called protamine.

Protamine, first extracted in 1874 by Friedrich Miescher from salmon sperm, is a strongly basic peptide [3,17]. It is already established in the therapy of diabetes mellitus as neutral protamine Hagedorn insulin [18] or cardiac surgery as an antidote against heparin overdoses [19]. It mainly consists of the basic amino acid arginine (up to 70%) [4,20]. These residues represent DNA-binding domains which explain the formation of DNA-protamine complexes in nature [3,17,20,21]. Researchers are making use of this property in developing carrier systems for DNAs and other oligonucleotides (ODNs). The so-called proticles, originally developed by Junghans et al. [22], are NPs formed by self-assembling in a nanotechnological “bottom-up” construction [23] and basically consist of protamine and an ODN. Therefore, due to the coacervation and nucleation processes, and as a function of their molecular recognition properties, one can say that the DDS builds itself [24]. Their easy manufacturing and a plethora of modification opportunities are making them very attractive [25,26,27,28,29,30]. Previous investigations with proticles including miRNAs—unfortunately—demonstrated one big obstacle which must be overcome. We have noticed that the strong electrostatic interaction between protamine and miRNAs leads to a lack of intracellular dissociation, and thus optimization is of paramount importance to improve miRNA functions. Hence, we strived for the strategy of tailoring the molecular interactions and creating newly ordered assemblies by adding citric acid (CA) to the binary proticle formulation. CA, which is a tricarboxylic acid, at a physiological pH represents a highly negatively charged small molecule which interacts with the basic arginine residues. The idea of supplementing proticles with CA has proven advantageous in the reduction of the strong electrostatic interactions between protamine and miRNAs. CA, as a universal plant and animal metabolism intermediate, also represents a non-toxic material with a natural origin and is applied in the pharmaceutical industry, as well as in the food and cosmetic industries [31]. Most commonly, CA is to be found in solid dosage forms as a pH modifier [32]. Researchers also described the implementation of CA as an excellent and harmless disinfecting agent against a variety of viruses (e.g., the human norovirus) [33]. Furthermore, functionalizing NPs with CA offers several advantages, such as a reduction in the agglomeration tendency, ameliorating stability in biological media, or an increase in their drug loading capacity [34].

Here, we highlight data that will focus on the proof of the destabilizing strategy as well as on the comparison of the binary proticles and the newly engineered ternary proticles in their physicochemical properties, as well as their in vitro characteristics.

### Functionalization Strategy and Nanoparticle Engineering

Crucial aspects of the functionalization strategy include the neutralization of the protamine molecules on one hand and the “protonation theory” on the other hand. Schneider et al. found that citrate molecules have strong attractive interactions with the guanidine group of arginine. In their work, they also revealed superior protein agglomeration suppression due to the newly formed arginine-citrate-salt (or in this work, protamine-citrate-salt). Furthermore, they demonstrated that citrate molecules form large hydrogen-bonded structures with the arginine molecules [35]. We hypothesized that a charge neutralization of protamine is achieved. This status is of crucial importance, because a reduced number of presented binding options is believed to go along with a lower miRNA binding efficiency and reduced NP stability. Another feature of this strategy is the salt-induced precipitation of proteins. It is well known that the addition of salt in watery solvents can provoke fractionation [36]. Kunz et al. offered an English translation of the original paper [37]. The increase in the salt concentration caused the attraction of water molecules by the salt ions. Consequently, the interaction between the charged parts of the proteins was reduced [38]. Thus, after the dissociation in a watery solvent, competition occurs between the opposite charged molecules or atoms. In this work, theoretically, protamine-citrate complexes are formed by combining CA and aqueous protamine solutions. These newly composed salts interact with the negatively charged miRNAs. Furthermore, the hydrogen competes with the formed protamine citrate for interaction with the miRNAs. Therefore, the electrostatic binding strength between protamine and the miRNAs should be reduced. Bye et al. published a detailed review on different discussed mechanisms for the interaction between proteins, small organic molecules, and salts, which may also influence our presented new DDS [39].

Moreover, we expected the CA hydrogen to advantageously increase the electrostatic repulsion of the remaining positive charges and the protamine molecules among each other. Furthermore, a weakening of the binding affinity between the components was suspected. Molar protamine:CA ratios from 1:1 to 1:20 were tested. Protamine comprises about 16–17 arginine residues which contribute to 16–17 positive charges. Therefore, a steady state or a charge balance should be achieved when using a molar ratio of 1:5 because one citrate molecule showed three negative charges. To conclude, lower ratios still have a predominant moiety of the presented positive charges, and ratios higher than 1:5 present a CA surplus.

## 2. Materials and Methods

For NP complexation, two different small, single-stranded, non-coding RNA molecules (miRNAs) were used, and all were obtained from Dharmacon (GE Healthcare, Lafayette, Colorado). The miRIDIAN^TM^ miRNA mimic negative control (UCACAACCUCCUAGAAAGAGUAGA—MW 14,074.31 g/mol) is a non-targeting negative control miRNA (ntcRNA) and therefore shows no physiological effects.

The second nucleic acid used to perform fluorescence experiments in vitro was the miRNA mimic transfection control with Dy547 (FluoNTC), including the sequence CUCUUUCUAGGAGGUUGUGA 5′ Dy547 (MW 14,566.9 g/mol). This miRNA was also used as a control miRNA and has no physiological efficacy.

To prepare the proticles, protamine free base (Grade IV, Histone-free) from Sigma Aldrich (Vienna, Austria) was used. CA anhydrate was purchased from Caesar & Loretz GmbH (Hilden, Germany), sodium citrate (SC) was obtained from Herba Chemosan (Vienna, Austria), and sodium hydroxide (NaOH) was obtained from Carl Roth GmbH + Co KG (Karlsruhe, Germany). Stock solutions well as dilutions were prepared in special RNase-free water (HiPerSolv CHROMANONORM^®^ for HPLC, VWR International, Darmstadt, Germany). Acetonitrile (HPLC gradient grade, VWR internationals, Radnor, PA, USA) and trifluoroacetic acid (for HPLC, VWR internationals, Radnor, PA, USA) were used for the HPLC measurements.

### 2.1. Particle Preparation

As was already mentioned, proticles are produced by self-assembling. Thus, the binary standard NPs were formed by combining aqueous solutions of miRNAs and protamine (mass ratio of 1:3), leading to a final miRNA concentration of 3.55 µM (=50 µg/mL). The dispersion was mixed, vortexed, and incubated for 15 min at RT. This procedure was also performed to prepare the functionalized ternary NPs, but before the NP formation was induced, the protamine solution was supplemented with CA to achieve molar ratios of 1:1, 1:2, 1:5, 1:10, and 1:20 (protamine:CA). These ternary proticles will be referred to as 1:1-NPs, 1:2-NPs, and so on. To assess our “protonation theory”, we also exchanged CA with sodium citrate (SC). All ratios as well as the measurement set-ups remained the same. To reveal the real impact of the acid ‘s hydrogen, dispersions with CA-NPs and SC were compared.

### 2.2. Encapsulation Efficacy

As a kind of quality check or “purification study”, the drug loading efficiency, as well as the quantification of the bound components, were determined by indirect quantification methods using a spectrometer (Eppendorf BioSpectrometer^®^ kinetic, Eppendorf AG, Hamburg, Germany) and reversed-phase high-performance liquid chromatography (RP-HPLC; Agilent 1260 Infinity, Agilent technologies, Santa Clara, California). The protamine and CA concentrations were evaluated with a Zorbax Bonus-RP 4.6 × 75 mm column (Agilent Technologies, Santa Clara, CA, USA). Mobile phase A was 0.05% trifluoroacetic acid (TFA), whereas mobile phase B was acetonitrile with 0.05% TFA. A gradient was used with a flow rate of 2.0 mL/min at 35 °C and an injection volume of 20 µL. The measurement started with 1% B for 1 min and increased to 30% B within 4 min, followed by 60% B for 2.5 min for cleaning. To detect the protamine and CA, a wavelength of 235 nm was used. The miRNAs were quantified on the spectrometer by performing a spectral scan from 226 to 350 nm. A 100% protamine solution in water (150 µg/mL) was used as a blank. A standard curve with stock solutions of each component was prepared previously. Before the measurements, the NP dispersions were centrifuged (Centrifuge 5804 R, Eppendorf, Hamburg, Germany) for 3 h at 20 °C and 20,000 rcf, and the supernatant was analyzed. The bound components were calculated as the difference between the amount of applied component and the detected value as a percentage.

### 2.3. Dynamic Light Scattering and Electrophoretic Light Scattering Experiments

The NP size, the particle size distribution (PSD), and the polydispersity index (PdI) were evaluated by dynamic light scattering (DLS) utilizing a Malvern Zetasizer Nano ZS (Malvern Instruments, Malvern, UK). All measurements were performed in a UV micro cuvette (UVette^®^ 220–1600 nm, Eppendorf AG, Hamburg, Germany) at 25 °C and in dual-angle mode (173° backward mode and 12.8° forward mode). The NP size was determined after 15 min (referred to as 0 h) and 3 h of incubation at RT. To measure the electrophoretic mobility of the particles, the surface charge (zeta potential) of the NPs was determined by electrophoretic light scattering (ELS). A clear folded capillary cell (Malvern Instruments, Malvern, UK) was used. All samples were diluted at a 1:10 ratio with water (pH 7, 50 µS) to a final volume of 700 µL. The pH values of the probes were evaluated with a pH meter (Lab 860, SI Instruments, Mainz, Germany).

### 2.4. Stability Assay

The functionalization strategy aimed to achieve a reduction in NP stability by CA supplementation. Thus, an easy-to-handle NaOH stability assay was established which was adapted from Lochmann et al. [40]. The proticles were treated with 0.01 M NaOH in a proticle dispersion to a NaOH ratio of 1:4. It was directed to disrupt the electrostatic attraction forces via protonation. Moreover, to consider dilution effects, control groups with RNAse-free water were measured too. The same procedure was applied to all samples, particle preparation was performed as already described, and NaOH or water was added. Subsequently, the samples were incubated and shaken on a Thermomixer comfort (Eppendorf AG, Hamburg, Germany) at 60 °C and 1000 rpm for 1 h. After the incubation period, the samples were allowed to cool down to RT for 15 min. Data were obtained via DLS, and the pH of all samples was traced to gain further information about the assay and the proticles. Hence, we expected a CA concentration-dependent loss in stability by applying the NaOH assay due to additional protonation. Data are presented as the percentage of NP stability after the treatment, and binary proticles are referred to as 100%.

### 2.5. Fluorescence Anisotropy

To further investigate the destabilizing effect of CA, the fluorescence anisotropy of fluorescently labeled proticles dependent of CA was measured. Measurements were performed in RNAse-free water with a Jasco FP-6500 spectrofluorometer (Jasco Inc., Pfungstadt, Germany) equipped with excitation and emission polarizers at an emission wavelength of 560 nm upon excitation at 525 nm. The slit widths were 5 nm for excitation and emission. The anisotropy signal of 5-µg/mL fluorescence-labeled RNA was recorded after 15 min of incubation at RT with pre-formed protamine-CA complexes of varying molar ratios.

The fluorescence anisotropy is defined as in Equation (1) [41]:

r = (I_VV_ − G × I_VH_)/(I_VV_ + 2G × I_VH_); −0.2 ≤ r ≤ 0.4 (1)

where I_VV_ is the fluorescence intensity recorded with excitation and emission polarizers in vertical positions and I_VH_ is the fluorescence intensity recorded with the emission polarizer aligned in a horizontal position. The G factor is the ratio of the sensitivities of the detection system for vertically and horizontally polarized light G = I_HV_/I_HH_.

### 2.6. Atomic Force Microscopy

While DLS delivers an overview of the proticle sizes in suspension, it cannot study particles individually, and assumptions about the shape as well as the refractive index are needed. The shape of the proticles is also of great importance to cellular uptake, as endocytosis has been shown to energetically favor spherical rather than elongated particles [42]. Atomic force microscopy (AFM) was employed to image individual proticles, although this method requires the proticles to be deposited on a surface, which in this case was highly oriented pyrolytic graphite (HOPG, grade ZYB). Immediately after preparation, the proticle suspensions were diluted in various amounts—1:49 for the results shown—with RNAse-free water, and 50 μL of the diluted suspension was then immediately deposited onto a 10 mm × 10 mm freshly cleaved HOPG surface in the laminar flow box to minimize contamination and covered in a Petri dish. After air drying for at least 24 h, the samples were imaged in air under atmospheric conditions using the intermittent contact mode (also called the tapping mode). The measurements were performed with a FlexAFM 5 from Nanosurf (Nanosurf AG, Liestal, Switzerland) controlled by a Nanosurf C3000 Controller on top of a Zeiss Axio Observer (Carl Zeiss AG, Oberkochen, Germany) with the corresponding C3000 software (version 3.10.0.26). The cantilevers were Tap300Al-G (BudgetSensors, Sofia, Bulgaria) which nominally had a resonance frequency of 300 kHz, a spring constant of 40 N/m, and a tip radius below 10 nm. Gwyddion was used for initial data treatment (supplemental 1) and image analysis (supplemental 2), while MATLAB was used for further data analysis.

### 2.7. Cell Culture

#### 2.7.1. In Vitro Transfection

Cell culture experiments were performed in mouse embryotic fibroblast-derived 3T3-L1 preadipocytes. The cells were cultivated under CO_2_ water-saturated atmosphere in a complete proliferation medium (PM). The PM consisted of low-glucose Dulbecco’s Modified Eagle’s Medium (lgDMEM) supplemented with 10% fetal bovine serum (FBS), 1% 4-(2-hydroxyethyl)-1-piperazineethanesulfonic acid (HEPES), 1% L-glutamine, and 1% penicillin/streptomycin. Transient transfections of proticles were performed in non-confluent 3T3-L1 preadipocytes. For this purpose, the cells were seeded at a density of approximately 8 × 10^3^ cells/well onto 96-well plates (Greiner Bio-One GmbH, Frickenhausen, Germany) for cytotoxicity and uptake studies 24 h before transfection and cultivated in complete PM. For live cell imaging, 7 × 10^4^ cells were seeded onto glass-bottom dishes (WillCo Wells B.V., Amsterdam, The Netherlands) 24 h before transfection and cultivated in complete PM.

#### 2.7.2. Cytotoxicity Assays

The cytotoxicity of single components, as well as binary and ternary proticles, was evaluated by applying the lactate dehydrogenase (LDH) assay (CytoTox-ONE^TM^;Assay) and the MTS (3-(4,5-dimethylthiazol-2-yl)-5-(3-carboxymethoxyphenyl)-2-(4-sulfophenyl)-2H-tetrazolium) assay (CellTiter 96^®^ Aqueous One Solution Cell Proliferation Assay). Both were ordered from Promega Corporation (Madison, WI, USA). Stock solutions were diluted using lgDMEM to final concentrations of 10, 50, 100, 250, 500, and 1000 nM. The procedure followed the provider’s manual’s instructions. Briefly, seeded cells were transfected with proticles and incubated at 37 °C for a period of 4 h. Afterward, 25 µL of the supernatant was transferred into a white 96-well plate (Greiner Bio-One GmbH, Frickenhausen, Germany) to perform the LDH assay. The residual NP dispersions were removed, and the remaining cells were washed and covered with serum-free lgDMEM. The MTS solution was added, followed by another incubation period of 4 h at 37 °C. The absorbance (excitation 492 nm) was measured by employing a CLARIOstar^®^ plate reader (BMG LABTECH, Ortenberg, Germany).

The LDH substrate was added to the separated supernatants. Next, the plates were incubated for 20–30 min at 37 °C. Afterward, the stop solution was added. The fluorescence signal was detected by utilizing the aforementioned plate reader (excitation: 455 nm; emission: 590 nm). In general, untreated cells were used as an MTS positive control, and lysed cells were used as an LDH positive control.

#### 2.7.3. Quantitative Cellular Uptake

Moreover, to gain information about the quantitative NP uptake, cells were transfected with binary and ternary proticles containing FluoNTC 24 h after seeding and incubated at 37 °C for a period of 4 h. Beforehand, proticle stock solutions were diluted with lgDMEM to a final nucleic acid concentration of 800 nM. After incubation, the cells were washed with PBS and covered with serum-free media, and the fluorescence signal (excitation: 455 nm; emission: 590 nm) was detected with a plate reader. Many other working groups applied similar methods to quantify the NP uptake in the cells beforehand [43,44,45].

#### 2.7.4. Live Cell Imaging Using Fluorescence Microscopy

In order to trace the fluorescently labeled CA-supplemented proticles after in vitro transfection, a fluorescent microscope was used (Zeiss Axio Observer, Göttingen, Germany). The uptake of the fluorescently labeled proticles was tracked over time (ca. 30 min, 2 h, and 3 h) in living cells at 37 °C. The classic binary proticles were used as a control. For in vitro transfection, both formulations were diluted using lgDMEM to a final nucleic acid concentration of 400 nM. The trafficking of the NPs in living cells was traced by using a live cell lysosomal dye: LysoTracker^®^Green (Thermo Fisher, Waltham, MA, USA). The LysoTracker^®^Green dye was prepared according to the manufacturer’s guidelines. Briefly, the dye was thawed and diluted using lgDMEM to a final concentration of 65 nM. Before in vitro transfection, the dye was mixed with an equal volume of CA or binary proticles. Thus, the final concentration of LysoTracker^®^Green applied was 32.5 nM.

The 3T3-L1 cells were incubated with the samples for 30 min at 37 °C. Afterward, the cells were washed using PBS and inspected under the fluorescent microscope at intervals of 30 min, 2 h, and 3 h. Cells treated using binary proticles were used in order to evaluate if CA had any influence on the uptake and trafficking of the NPs. Cell-only (untreated cells) samples were used as a further control. The FluoNTC was detected at a 543-nm excitation wavelength. LysoTracker^®^Green was detected at a 504-nm excitation wavelength.

## 3. Results

### 3.1. Encapsulation Efficacy

**Figure 1:**
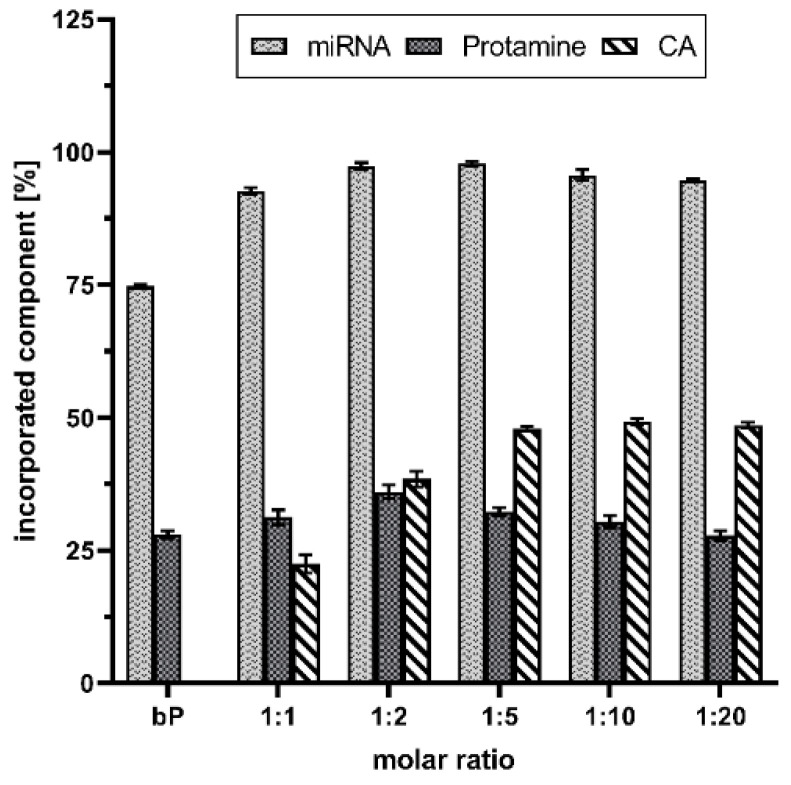
microRNA (miRNA) loading, as well as quantification of administered excipients of citric acid (CA), supplemented proticles, and binary proticles (bP). Data are presented as mean values (*n* = 3) ± standard deviation. The results were obtained by indirect quantification, measuring the supernatants of the nanoparticle dispersions after centrifugation, and calculating the difference between the applied amount and the detected amount as a percentage. The molar ratio refers to a molar protamine:CA ratio.

To gain quantitative information about the formulations and make a kind of quality-check, spectrophotometric and RP-HPLC measurements were performed. As highlighted in Figure 1, CA increased the drug load. More than 90% of the miRNAs were packed in the advanced proticles independent of the CA concentration. In comparison, the miRNA loading capacity of the binary proticles was about 75%. As expected, the chosen miRNA:protamine mass ratio of 1:3 resulted in 30% bound protamine in the proticles. The highest amount of protamine was found in the 1:2 NPs (38 ± 1.5%). Absolutely interesting results were obtained for determining the CA concentration in the proticles. The data are expressed as a percentage of the applied CA amount of each ratio. It can be seen that up to the molar ratio of 1:5, a permanent increase is noticeable. When comparing the three highest CA ratios, this trend continued. Independent from the applied amount of CA, about 50% were detected, which correlated with the increasing CA concentrations. These measurements represent the successful implementation of CA in binary proticles. This further demonstrates that protamine alone is capable of binding the miRNAs properly, and CA supplementation improved the quality of the DDS.

### 3.2. Dynamic Light Scattering and Electrophoretic Light Scattering Experiments

**Figure 2:**
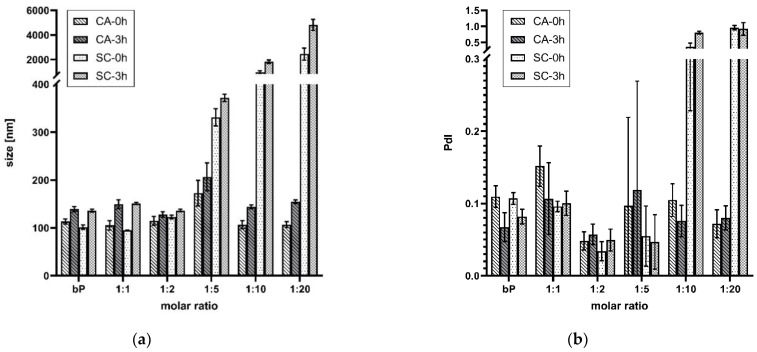
(**a**) Mean particle size throughout 3 h. (**b**) Mean particle size distribution throughout the 3 h. Comparison of proticles supplemented with citric acid (CA) and sodium citrate (SC). The measuring points at 0 h represent 15 min and 3 h after preparation, and the results are given as mean values (*n* = 3) ± standard deviation. The molar ratio refers to a molar protamine:CA ratio.

DLS was applied to check the particle size and PDS. Time-dependent measurements were performed 15 min (0 h) and 3 h after incubation at RT. The final proticles contained an miRNA concentration of 3.55 µM. Figure 2a displays the comparison of data of the classic binary proticles, CA proticles, and the control group proticles supplemented with SC. The results imply that the samples incorporating lower CA concentrations than 1:5 had no noticeable influence on particle growth in comparison with the binary proticles. Their values varied between 105 nm and 115 nm at 0 h. After 3 h of incubation, however, an increase was observed for the binary proticles and the CA-NPs (128–149 nm). Furthermore, all these samples show monodispersed distribution which correlated with a value < 0.2. Interestingly, the 1:5 NPs showed a remarkable increase in size (173 ± 43 nm, 0 h–207 ± 29 nm, 3 h). Higher CA concentrations did not differ from the 1:1 NPs and 1:2 NPs neither after 0 h nor after 3 h. Moreover, the replacement of CA with SC highlighted divergent results. The samples with lower SC ratios than 1:5 were not distinguishable from the other test groups, but a very drastic increase in NP size was detected at higher concentrations. Fascinatingly, the NPs incorporating SC seemed to have strong agglomeration tendencies, applying a SC ratio of 1:10 where the values were >1800 nm and even higher (>2000 nm) when raising the SC concentration. Additionally, a very polydispersed distribution was measured for both (Figure 2b). After 3 h, agglomeration continued, which revealed a clear indicator that higher concentrations of SC as a supplement were not appropriate for proticle improvement. 

**Figure 3:**
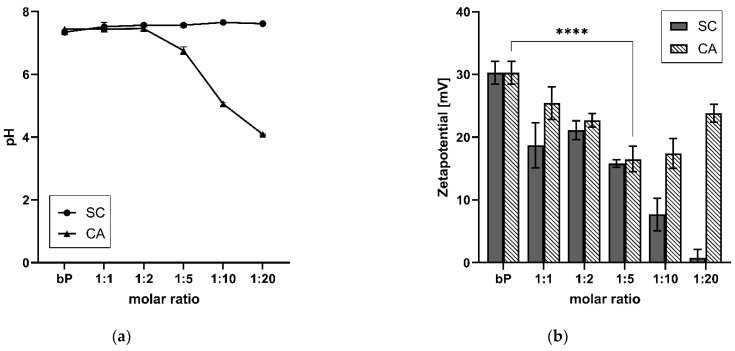
(**a**) Comparison of the influence of citric acid (CA) and sodium citrate (SC) on the pH of ternary proticles. (**b**) Zeta potential of CA- and SC-loaded ternary proticles in comparison to binary proticles (bP). Data are shown as mean values (*n* = 3) ± standard deviation. It can be seen that applying CA at a molar ratio of 1:5 led to a highly significant (**** *p* < 0.001) decrease in the zeta potential. The molar ratio refers to a molar protamine:CA ratio.

Another parameter that exhibited the influence of CA on the proticles was the pH. As depicted in Figure 3a, the pH levels of the proticle dispersions with CA and SC were determined in comparison with the classic binary proticles. Binary proticle dispersions have a neutral pH of about 7.35. As expected, the same pH could be detected for all samples supplemented with SC. They did not show any differences within this test group. On the contrary, CA did demonstrate a pH shift. The critical point of the DDS was the ratio of 1:5. The dispersions of the 1:1 NPs and 1:2 NPs had a neutral pH (pH 7.4), and with increasing CA moieties, the acidic shift began. Thus, around the highest CA concentration, the pH dropped to 4.01.

The zeta potential is a parameter that expresses the surface charge and colloidal stability of the nanoparticulate DDS. Moreover, the binary proticles showed a surface charge of about 30 mV (Figure 3b). Due to CA supplementation, a decrease in the zeta potential, and thus a destabilizing tendency, was detected. The fivefold surplus once more highlighted the critical point of the DDS (16.5 ± 2 mV) and resulted in a highly significant (*p* < 0.001) reduction in the zeta potential. Higher CA ratios demonstrated a renewed rise in zeta potential but remained lower than the binary proticles (23.8 ± 1.4 mV). This rise probably stemmed from the acid surplus, which increased the protonation of the NPs. On the contrary, it was discovered that the replacement of CA with SC led to a complete loss in colloidal stability. Until a ratio of 1:5, the results were quite similar to the CA proticles but were followed by a big drop close to zero (0.8 ± 1.3 mV).

### 3.3. Modified Stability

**Figure 4:**
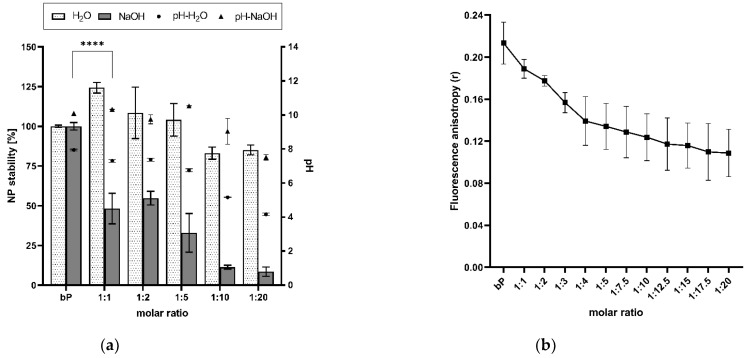
(**a**) Results of the NaOH stability assay and pH level evaluation. A highly significant (**** *p* < 0.001) reduction in nanoparticle stability was observed. With increasing citric acid concentrations, the pH decreased. (**b**) Fluorescence anisotropy (r) in correlation with increasing citric acid concentrations. Due to supplementation with citric acid, the anisotropy of the functionalized proticles decreased, which indicates a reduction in binding affinity between the microRNAs and the protamine-citric acid complex. The molar ratio refers to the molar protamine:CA ratio.

The modified stability of the proticles was determined by treating the NP dispersions with 0.01 M NaOH and applying DLS. Comparing the results of the CA-NPs and the binary proticles treated with NaOH and water, a strong decrease in NP stability was revealed due to NaOH addition. Our functionalization strategy led to a reduction of up to 92% in comparison with the binary proticles. These effects are highlighted in Figure 4a, and the proticles without CA supplementation were considered to be 100% for the water as well as the NaOH set-up. Envisaging the water group results, an interesting trend could be scrutinized. Low CA concentrations seemed to improve NP formation even if they were diluted with water. Only the CA ratios of 1:10 and 1:20 showed a slight decrease, resulting in an NP stability of about 85%. Completely different observations were made in the NaOH group. These data imply a highly significant (*p* < 0.001) decrease due to CA, as a reduction of about 50% was determined relating to the lowest CA concentrations (1:1 and 1:2). Higher deviations around 1:5 NPs indicated once more the critical state of the advanced NPs at this ratio. Still, the NP stability was decreasing. It is interesting to track the deprotonating effect of NaOH, as it seemed to be increasing or even showing higher efficacy at a protamine:CA-ratio of 1:5. There is the growing realization that the ionic interaction between the miRNAs and the protamine-CA complex was hampered. Moreover, NaOH made the impression to possess the ability to buffer the acid function, which—as was already demonstrated—was decisive for our NPs. These observations were in good accordance with the pH measurements. No shifts in pH were noticed until this ratio. Here, the functionalization of proticles with higher ratios of CA (1:10 and 1:20) demonstrated the successful decrease in NP stability. For these samples, about 10% of NP stability was evaluated, which correlated to complete complex disruption. Taking the pH measurements into account, the already demonstrated gradient was visible again. In comparison with the water group, an alkaline shift was found. One objective of this research was to decrease the electrostatic strength between the protamine and miRNAs by functionalizing these proticles with CA. Through this NaOH stability test, we indicated the outperforming loss in stability of the engineered NPs in comparison to the classic binary proticles. Next to this conclusion, further parallels can be seen when considering the zeta potential results again. The NPs functionalized with SC had no hydrogen, and thus the zeta potential was constantly going down. The same stepwise decrease pattern can be observed when deprotonating CA-NPs with NaOH (comparing Figure 4a and Figure 3b). Hence, in both ways, our destabilization hypothesis could be supported.

To further analyze CA-induced proticle destabilization, we measured the influence of CA on proticle assembly by fluorescence anisotropy. This technique can be used to detect molecule interactions depending on changes in the rotational mobility of a fluorophore covalently attached to a binding partner. As shown in Figure 4b, the fluorescence anisotropy signal derived from the interaction of protamine and Dy547-labeled miRNAs dropped into a saturable mode, depending on increasing CA concentrations. As the fluorescence anisotropy signal is sensitive to size variations of a molecule complex, we deduced that the observed signal decrease was derived from the ability of CA to weaken the protamine–miRNA interaction and, consequently, proticle assembly as well as NP stability. These results correlate with the overall particle destabilization observed by the aforementioned stability assay.

### 3.4. Atomic Force Microscopy

**Figure 5:**
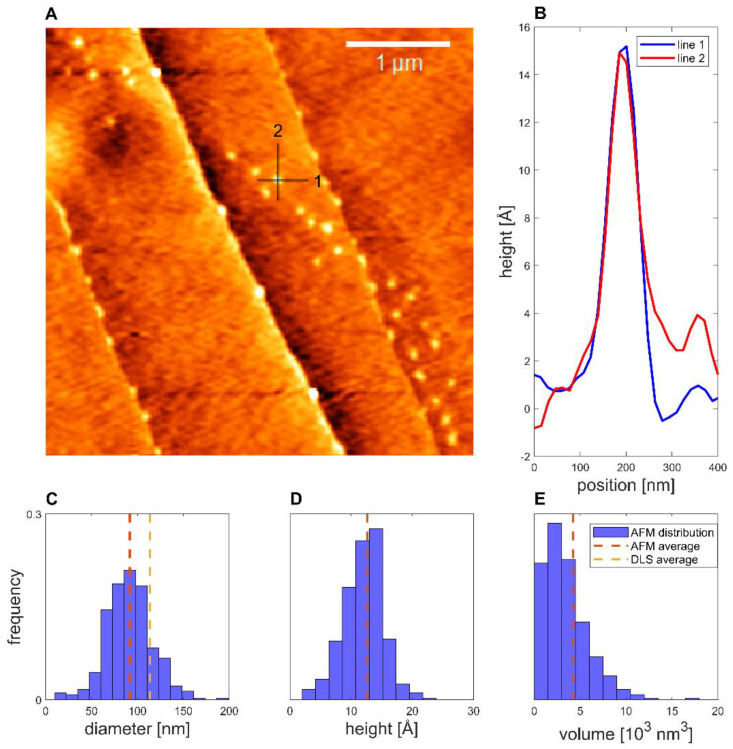
Binary proticles deposited on HOPG. (**A**) Topographical image, with the colors representing height. (**B**) Example of line profiles of a proticle. (**C**) Equivalent diameter distribution. (**D**) Height distribution. (**E**) Volume distribution (*N* = 358).

The deposition of binary proticles with no CA was found to follow lines on the HOPG surface, as highlighted in Figure 5. Imaging the NPs showed that the average equivalent diameter of the proticles was 92 ± 1 nm, while the average height was 12.6 ± 0.6 Å and the average volume was (4.2 ± 0.8) × 10^3^ nm^3^. This volume was only 0.6% of the volume inferred from the DLS data, which indicates that the dispersed proticles represented a very floppy and hydrated construct. Furthermore, a spherical shape could be demonstrated.

**Figure 6:**
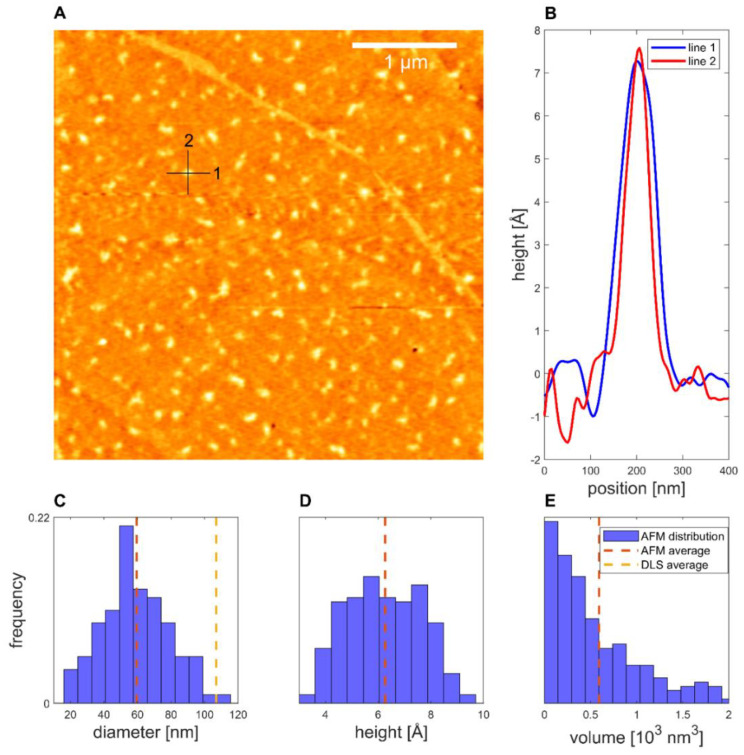
The 1:20-NPs deposited on HOPG. (**A**) Topography. (**B**) Line profiles of a proticle. (**C**) Equivalent diameter distribution. (**D**) Height distribution. (**E**) Volume distribution (*N* = 200).

Representing the CA formulation, the proticles with the highest CA molar ratio (1:20) were evaluated. For the 1:20 NPs, a smooth distribution of structures was found for the 1:49 dilution (Figure 6). The average diameter of the proticles was 60 ± 1 nm, while the average height was 6.3 ± 0.1 Å and the average volume was (1.49 ± 0.08) × 10^3^ nm^3^. This volume was only 0.09% of the volume inferred from the DLS data. Thus, it is arguable that CA supplementation increases the hydration level of the DDS even more, even if the volume of both formulations is very low. As highlighted, the shape of the CA-supplemented proticles was not as clearly defined as it was for the binary proticles. Their appearance was very diverse, and the particles were more elongated than spherical.

**Figure 7:**
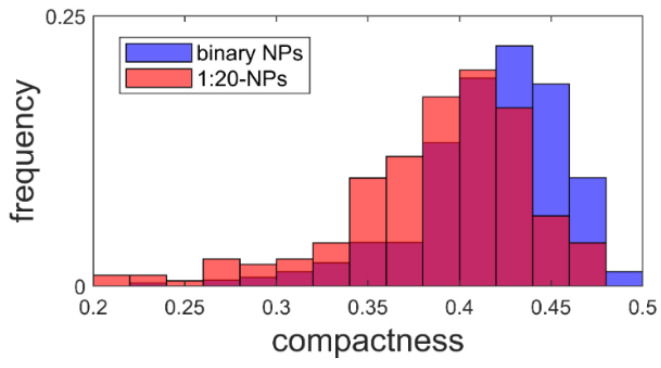
The compactness of proticles with (*N* = 200) and without (*N* = 369) citric acid supplementation.

The shape of the measured structures can be described by the compactness C, given in Equation (2):

(2) $$C=\frac{4\pi A}{P^{2}}$$

where P is the perimeter and A is the area. This results in a value between zero and one, where one is the compactness of a perfect circle [46]. The calculated compactness (Figure 7) dropped by 8% from 0.422 ± 0.003 to 0.388 ± 0.004 when using CA.

### 3.5. Cell Culture

#### 3.5.1. In Vitro Cytotoxicity Assay

**Figure 8:**
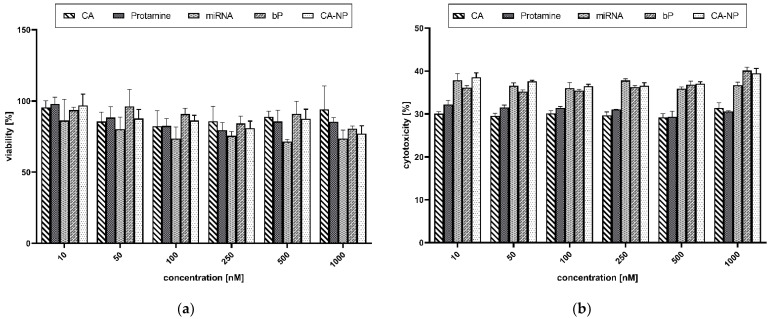
(**a**) MTS viability assay. (**b**) LDH cytotoxicity assay. Data are expressed as mean values (*n* = 3) ± standard deviation. Here, 100% viability was correlated with untreated cells, while 100% cytotoxicity was correlated with cells treated with a lysis buffer. CA: citric acid; miRNA: microRNA (non-targeting control microRNA); bP: binary proticles; CA-NP: citric acid-supplemented proticles.

Metabolically active cells are capable of transforming MTS tetrazolium compounds (Owen’s reagent) into a colored formazan. The measured absorption of the brownish dye presumably accomplished by dehydrogenases was correlated to cell viability. The untreated cells were considered 100% viable. The screening showed high cell viability (>70%) and even a slight reduction in cytotoxic potential due to NP formation (Figure 8a).

The cellular toxicity was scrutinized by applying an LDH toxicity assay. Often, the integrity of the cell membrane is used to define cell viability. If the membrane is disrupted, released components from the cytoplasm are leaking into the medium. This is a trustworthy parameter for estimating the moiety of non-viable or disrupted cells. Lysed cells were considered 100% cytotoxic. As visible in Figure 8b, an LDH release of about 30–40% was determined. Thus, damage to the cell membrane was noticeable. Interestingly, the cells transfected with the naked miRNAs showed the highest toxic potential and the lowest metabolic activity.

It is also worth mentioning that both assays demonstrated no concentration dependency. Nearly the same results were obtained when applying low concentrations (10 nM) in comparison with the highest concentration of 1000 nM.

#### 3.5.2. Quantitative Cellular Uptake

**Figure 9:**
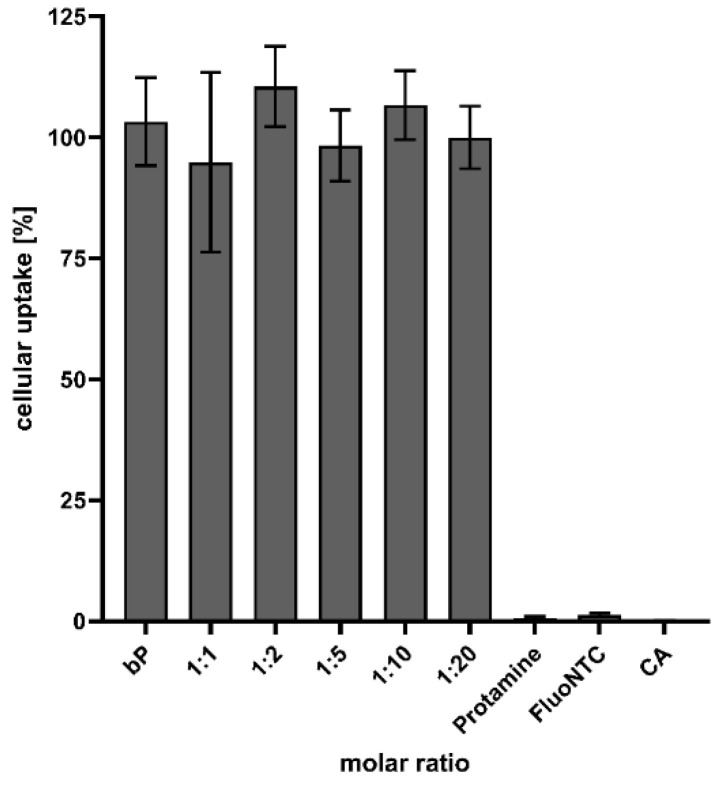
Quantification of proticles and CA-NP uptake. Data are presented as mean values (*n* = 6) ± standard deviation and are expressed as a percentage of the uptake, where the uptake of binary proticles (bP) is considered to be 100%. FluoNTC: fluorescence-labeled non-targeting control microRNA; applied FluoNTC concentration = 800 nM. The molar ratio refers to a molar protamine:CA ratio.

The cells were treated with fluorescence-marked NPs to quantify and determine the transfer efficiency. After an incubation period of 4 h, and under serum-free conditions, the signal was quantified utilizing a plate reader. As highlighted in Figure 9, particle formation is of major importance to achieve successful drug delivery and cellular uptake. Nearly no signal could be detected when transfecting cells with naked FluoNTC, but the treatment with proticles resulted in a thriving fluorescence signal. Furthermore, no difference between the CA-NPs and the classic proticles could be seen.

#### 3.5.3. Live Cell Imaging Using Fluorescence Microscopy

**Figure 10:**
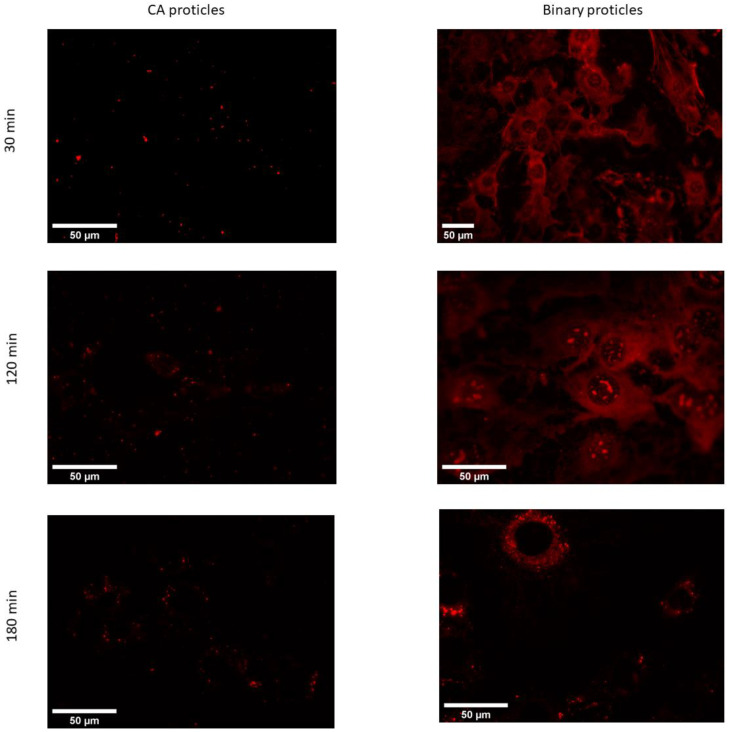
Fluorescence microscopic images of living 3T3-L1 cells at 37 °C transfected with CA-proticles (protamine:CA molar ratio of 1:10) and binary proticles (red) at 400 nM (30 min, 120 min, and 180 min post-transfection).

The obtained microscopic images show that the uptake of CA-NPs (for demonstration reasons, we used a molar ratio of 1:10) occurred within the first hour of incubation with the cells (Figure 10). At the very beginning of incubation, the particles tended to localize inside the cytoplasm and the nuclei of the cells. The signal coming from the cytoplasm seemed to be both diffusely spread around and also organized in a vesicular manner. This could suggest that uptake partly occurs via one of the endocytic pathways, while the diffusely spread signal can be due to direct uptake.

**Figure 11:**
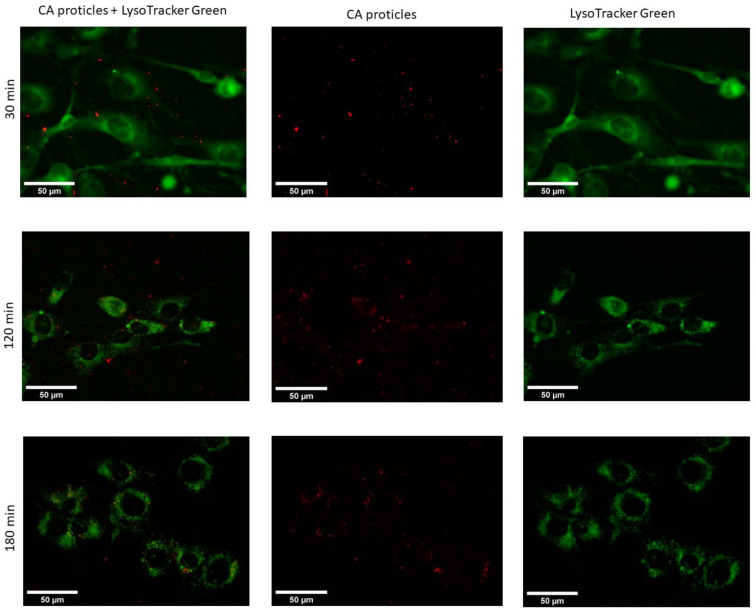
Fluorescence microscopic images of living 3T3-L1 cells at 37 °C transfected with CA-proticles (protamine:CA molar ratio of 1:10) (red) at 400 nM (30 min, 120 min, and 180 min post-transfection). LysoTracker^®^Green was used to stain the lysosomes inside the cells.

In addition, when compared with the proticles in terms of intracellular localization and trafficking, the CA-NPs did not show a big difference. Nevertheless, the situation changed two and three hours after transfection (Figure 11). At these time points, we could observe that the fluorescent signal was located exclusively in the cytoplasm with no residue in the nucleus. What is more interesting in this case is that the signal was no longer diffuse but located in the vesicles. When it comes to cellular trafficking, from the images obtained using the LysoTracker^®^Green, it seems that the greater part of CA-NPs does not colocalize with lysosomes inside the cells, and this situation does not change over time.

## 4. Discussion

By supplementing binary proticles with CA, a reduction in NP stability should be provoked. To evaluate the influences of the third component on the nanoparticulate DDS’s properties and to figure out if the functionalization strategy succeeded in decreasing the stability of the newly developed ternary proticles were the objectives of this work. Characterizing the physicochemical properties of functionalized proticles highlighted the differences between the classic and the newly designed proticles. In order to reveal the influence of the CA hydrogen, proticles incorporating CA and SC were prepared. In general, over nearly all performed measurements, it was noticeable that the samples containing supplements at a molar ratio of 1:5 acted differently. This phenomenon can be explained by the equilibrium of the charges between protamine and the citrate molecules. A critical point of neutralization was achieved, and consequently, the repulsion between the individual NPs decreased due to the indifferent charges of the NPs. In this state, the DDS is at an unstable balance, which is visible in the severe deviations. As was already mentioned, protamine incorporated about 16–17 positively charged arginine molecules. Therefore, at the 1:5 ratio in particular, a charge neutralization could occur due to 5 applied citrate molecules which were triple negatively charged. It seems that until a molar ratio of 1:5, only the citrate molecules affected the particles because both the CA and SC formulations behaved similarly. Thus, it seems that this ratio was identified as the significant bottleneck of the modified proticles. As these happenings occur nearly everywhere, they will not be discussed in detail in this chapter again.

Applying spectrophotometry and RP-HPLC enabled the quantification of our formulation and demonstrated the successful encapsulation of miRNAs. We noticed that our functionalization strategy improved the drug load. The overall view of these measurements allowed the following assumption to be drawn: As CA supplementation provoked a slight increase in protamine binding capacity, an increase in drug loading seems to be the logical result. The persistent increase in bound CA correlated to increasing concentrations is arguable by the huge surplus of protamine and the favorite attraction of the citrate molecules to the arginine. Therefore, as long as arginine residues—provided by protamine—are available, the citrate molecules will electrostatically bind to them. Thus, they are not detectable anymore in the supernatant. Additionally, as already referred to in the literature and demonstrated in the AFM measurements, we could see that CA supplementation increased the level of hydration. These formed “sponges” or “clouds” are loose complexes allowing single molecules to linger and interact with others. This fact provides another explanation for the increased drug load in comparison with binary proticles.

DLS measurements were perfumed to gain information about the NP size and PDI. No noticeable influence on the particle size when comparing the binary and ternary proticles could be found until the molar ratio of 1:5. At this point, an increase in size was visible. However, CA concentrations higher than 1:5 caused a size reduction again. Conversely, when applying higher SC concentrations, the size increased drastically. Taking these observations into account, we assumed that the surplus of CA encouraged repulsion, and the aforementioned “protonation theory” was successful. Due to their interparticulate repulsive force, the NPs showed a lower agglomeration tendency, and thus the particle size decreased. CA replacement with SC supports this hypothesis because these NPs agglomerated immensely. The lack of hydrogen seems to even support the adhesion of the NPs among each other. Additionally, these findings are according to the literature. Schneider et al. revealed superior protein agglomeration suppression of the salt formation between the protamine and citrate molecules [35].

The zeta potential offers information about the colloidal stability and the surface charge of the NPs. A positive zeta potential could be detected for all tested groups: the CA-NPs as well as the SC-NPs and binary proticles. However, it has to be mentioned that their performance completely varied from each other. The zeta potential of the CA-NPs decreased significantly until the fivefold CA surplus and subsequently increased afterward. These results show several interesting points. On one hand, due to functionalization, it was possible to lower the colloidal stability, and on the other hand, the impact of the hydrogens on the surface charge could be determined. As already mentioned, increasing the CA concentration provoked a surplus of hydrogen in the dispersions, which further resulted in a new increase in the zeta potential. Contrary results were found regarding SC-NPs. A clear concentration-dependent loss in surface charge was determined. The lack of hydrogen as well as the presence of only sodium ions, which are not able to provide protamine with more positive charges, explain this strong destabilization phenomenon. To summarize, through the zeta potential measurement, we could highlight a decrease in surface charge and colloidal stability due to functionalizing NPs with CA. Additionally, we would like to mention that these findings are in total accordance with the pH measurements.

Moreover, to detect the modified CA-proticle stabilization, a NaOH stability assay and fluorescence anisotropy measurements were performed. NaOH is a well-known substance for dissociating NPs by providing non-physiological, highly protonated conditions [40]. A pH-dependent dissociation and the change in ionic strength are the foundation of this assay. All functionalized NPs experienced a drastic loss in stability when treated with 0.01 M NaOH. Even the lowest CA concentration led to a highly significant (*p* < 0.001) destabilization in physiological pH. Furthermore, a beginning acidic pH shift could be observed when measuring the 1:5 NP sample. The critical state of the functionalized proticles could be reflected again. The following CA surplus led to complete complex destabilization. Interestingly, a decrease in NP stability occurred in the water control group too. One explanation for this phenomenon could be that high CA concentrations lead to a general increase in the total number of manufactured NPs. The repulsion between the positive protamine charges and the hydrogens complicates the formation process. Additionally, suppressed agglomeration tendencies due to CA were reported [35]. The applied fluorescence anisotropy measurements supported these findings. It can be suggested that the coacervation process, and therefore the self-assembly, was modified due to CA, which provoked the reduction in binding affinity.

Taking the AFM measurements into account, we noticed that for both AFM samples, over 200 structures with a diameter similar to the DLS values were observed. This made it plausible to identify these structures as proticles. However, since the NPs had to be deposited and dried before measurement, this likely caused some change in the proticles themselves. Likewise, the same proticle preparation method might yield slightly different proticle sizes. Only with this in mind can one properly compare DLS and AFM values. Both proticle formulations demonstrated such small NPs that they did not show a clearly defined edge, making it difficult to measure their diameter (supplemental 2), but one would expect the measured diameters to be overestimated due to the finite size of the tip [47]. Therefore, the diminished diameter when dried is likely real. The apparent height measured with AFM is known to be underestimated [48]. This is often explained as tip compression, meaning that the force exerted by the tip when measuring caused the structures to be flatter than they really were [47]. For instance, DNA heights have been measured to be 68% (1.36 nm) [48] and down to even 35% (0.7 nm) [49] of the true height (2 nm) [50], although more extreme deviations have been theoretically proposed [48]. For the binary and CA proticles, the measured heights were 11% and 6%, respectively, of the DLS diameters. This is much smaller than what is usually attributed to tip compression, and it is therefore unlikely that this is merely an artifact. Additionally, since the measured heights are close to those of DNA, the structures are likely only one or two layers of molecules thick, indicating that the proticles consist of a very small number of molecules. Studies using softer cantilevers or non-AFM methods might shed further light on this question. The reduced diameter and height suggest that both types of proticles shrink significantly when dried. If one corrects for a compression of 35%, the water content when hydrated is still >98%. As already mentioned, it has been postulated that the citrate molecules form large hydrogen-bonded structures with the arginine residues [35], and this might explain the huge water content. The proticles with CA exhibited a more irregular appearance, which was confirmed quantitatively with the compactness parameter, although the deposition process and the image resolution could influence the compactness. One interpretation of the lower compactness—taken together with the diminished size—is that the proticles are more loosely bound when they contain CA.

Cell viability was investigated by applying MTS and LDH toxicity tests. The results clarified that the naked miRNAs reduced the cell viability the most in comparison to the tested NPs. These results follow the outcome of the LDH toxicity assay, which evaluates the damage to the cell membrane. It is well known that negative surface charges can negatively affect cells and lead to cell membrane damage [51]. When complexing the components into NPs, the cellular tolerance seems to increase. Furthermore, we assume that the detected toxic effects are reversible. As was already described, the LDH assay was performed before the MTS assay and thus represented a shorter time window. Cell recovery was noticed, and the high metabolic activity of the cells transfected with NPs and single substances was evaluated via the MTS assay.

Furthermore, when it comes to the discrepancy between the MTS and LDH results, this might be because cell-penetrating peptides tend to cause mild membrane disruptions while entering cells, as well as cytoskeletal reorganization by activating the Ras/Rac GTP-ases when interacting with extracellular glycosaminoglycans. These mild disruptions are temporary and usually occur during the initial moments of incubation, after which the cells completely recover [16]. We also checked the membrane integrity via optical microscopy (data not shown). The pictures highlight an intact membrane without disruptions. However, first and foremost, we want to emphasize the general high cell viability and low toxic potential of all applied ingredients and NPs.

The intracellular quantification of proticles showed no difference in the binary and ternary DDSs. Thus, no negative effect due to functionalization on cellular uptake could be found. Of course, one always has to have in mind that this measurement gives no information about the localization of the NPs, as they can still just be associated on the surface of the membrane or be in the membrane. However, the widefield fluorescence microscopy results showed that the interaction of proticles and the cellular membrane happened fast. This interaction is the first step toward cellular internalization and is thought to be facilitated by the components of the extracellular matrix (ECM), glycosaminoglycans, and proteoglycans. These molecules serve as signaling molecules that can facilitate cellular uptake [16]. The strong interaction with the ECM is driven by the arginine-rich structure of protamine and specifically its guanidine groups, which facilitate transport through the membrane [52]. Arginine’s guanidine group is responsible for forming bidentate hydrogen bonds with sulfate and phosphate groups found on the cells’ surface, leading up to hydrophobic complexes that can help with the surface accumulation of proticles and their subsequent internalization. Furthermore, once inside the cells, the arginine-rich sequence (the so-called nuclear localization signal (NLS)) drives the proticles near the nuclear environment, which is logical, keeping in mind that protamine is known as a DNA-organizing protein [53]. Our unpublished studies further show that fluorescently labeled protamine (not complexed with miRNAs) also tends to internalize in cells. This leads us to believe that proticles are entering cells and not just settling at the bottom of the well. Moreover, confocal microscopy with fluorescent labeled proticles was performed before. Dinauer et al. also demonstrated successful NP uptake and highlighted the importance of proticles as potent DDSs [54]. Hence, this is another indication to support our hypothesis.

When it comes to the uptake route, it is still difficult to draw conclusions. However, the possibility exists that arginine-based nanocarriers exploit endocytic routes to enter cells, and this can also be one mode of entry for proticles [55]. Further studies using specific endocytic inhibitors would greatly improve our understanding of the intracellular fate of both binary and citric acid proticles.

## 5. Conclusions

In conclusion, there is growing evidence that the functionalization of proticles with citrate molecules does not follow the “repulsion theory” but rather supports particle growth from the point of a steady state, which correlates with higher citrate concentrations. Obviously, the repulsion effects due to the acid’s hydrogen are stronger than the citrate effects when comparing CA-NPs and SC-NPs. Additionally, the “protonation theory”, referring to the necessity of present hydrogens to have an adequate DDS, was also supported by the obtained data. The supplementation of proticles with CA further led to a drastic decrease in NP stability and binding affinity, which highlights the success of our functionalization strategy. Due to proticle engineering, it was possible to increase the drug load and therefore provide a DDS of high potential. A low cytotoxicity as well as efficient cellular uptake were demonstrated in the cell culture model. Moreover, two different uptake routes were discussed. One can say that these easy and quickly manufactured advanced proticles show great potential for future studies and help to add another piece to the puzzle of successful miRNA delivery.
